# *Protoparvovirus* Cell Entry

**DOI:** 10.3390/v9110313

**Published:** 2017-10-26

**Authors:** Carlos Ros, Nooshin Bayat, Raphael Wolfisberg, José M. Almendral

**Affiliations:** 1 Department of Chemistry and Biochemistry, University of Bern, 3012 Bern, Switzerland; 2 Centro de Biología Molecular “Severo Ochoa”, Consejo Superior de Investigaciones Científicas-Universidad Autónoma de Madrid, Cantoblanco, 28049 Madrid, Spain; nbayat@cbm.csic.es; 3 Copenhagen Hepatitis C Program (CO-HEP), Department of Infectious Diseases and Clinical Research Centre, Hvidovre Hospital and Department of Immunology and Microbiology, Faculty of Health and Medical Sciences, University of Copenhagen, 1165 Copenhagen, Denmark; rwolfisberg@sund.ku.dk

**Keywords:** parvovirus, *Protoparvovirus*, icosahedral capsid, receptor binding, capsid rearrangements, endosomal escape, trafficking signals, nuclear import, virus entry

## Abstract

The *Protoparvovirus* (PtPV) genus of the *Parvoviridae* family of viruses includes important animal pathogens and reference molecular models for the entire family. Some virus members of the PtPV genus have arisen as promising tools to treat tumoral processes, as they exhibit marked oncotropism and oncolytic activities while being nonpathogenic for humans. The PtPVs invade and replicate within the nucleus making extensive use of the transport, transcription and replication machineries of the host cells. In order to reach the nucleus, PtPVs need to cross over several intracellular barriers and traffic through different cell compartments, which limit their infection efficiency. In this review we summarize molecular interactions, capsid structural transitions and hijacking of cellular processes, by which the PtPVs enter and deliver their single-stranded DNA genome into the host cell nucleus. Understanding mechanisms that govern the complex PtPV entry will be instrumental in developing approaches to boost their anticancer therapeutic potential and improving their safety profile.

## 1. Introduction

The *Parvoviridae* is a family of small, non-enveloped, icosahedral viruses with linear single-stranded genomes, infecting an exceptionally wide range of animal hosts, from invertebrates to mammals, including humans. Currently, species within the genus *Protoparvovirus* (PtPV) of the *Parvoviridae* include [1]: *Rodent protoparvovirus 1* (H-1 parvovirus; H-1PV, Kilham rat virus, LuIII virus, minute virus of mice (MVM), mouse parvovirus, tumor virus X, rat minute virus); *Rodent protoparvovirus 2* (rat parvovirus 1); *Carnivore protoparvovirus 1* (canine parvovirus (CPV) and feline panleukopenia parvovirus (FPV)); *Primate protoparvovirus 1* (bufavirus) and *Ungulate parvovirus 1* (porcine parvovirus (PPV)). The diseases caused by PtPVs in their natural hosts range in severity from subclinical to lethal infections, depending on the virus and host factors such as age, immune-competence and health conditions [2].

The PtPV single-stranded DNA (ssDNA) genome, of approximately 5 kb in length, is organized in two open reading frames, a non-structural (NS) and a structural (VP) [3]. Structure and biophysical properties of PtPV capsids are of paramount importance to prevail in nature and enter their host cells. The parvovirus capsid is a 25 nm-diameter assembly of 60 subunits in a *T* = 1 icosahedral symmetry of two to four largely overlapping proteins commonly designated VP1 to VP4 [4]. The 3-D atomic structure of PtPV capsid is available for some virus members (e.g., [5,6,7]). Common features are the folding of their protein subunits into an eight-stranded antiparallel β-barrel topology, a β-cylindrical projection encircled by a canyon-like depression that surrounds the fivefold symmetry axes, and a pore at the center of the β-cylinder running between the surface and the interior of the capsid. The topology of the capsid surface differs among the parvoviruses conferring characteristic virus host range. Flexible domains in the capsid are the N-terminal sequences of the VP subunits, which are dispensable for virus assembly, but strictly required for infection, as they contain functional motifs that can be hidden or exposed on the capsid surface at specific host cell compartments to mediate virus entry [8,9,10,11] and egress [12].

The biological relevance of PtPVs is not only due to their pathogenicity in their natural host, but also because some rodent PtPVs, such as H-1PV, MVM and LuIII, display natural oncotropism and marked oncolytic activities against human cancer cells, while being non-pathogenic for humans. Parvovirus replication is strongly dependent on host cell factors expressed during the S-phase, due to the limited coding capacity of the genome and their inability to induce resting cells to enter S-phase [13,14]. Moreover, the virus cycle, particularly nuclear translocation of the structural subunits and capsid assembly, are tightly coupled to the cycle of the host cell [15]. The dependency of PtPVs on transformed-dependent factors [16,17,18,19] contributes to the high susceptibility of certain human tumor cells to PtPV-induced cell killing. Therefore, these features, and the low prevalence of pre-existing antibodies, make the group of rodent PtPV viruses promising tools to treat human tumors [20].

During the entry process, the robust PtPV virion must undergo defined structural changes and overcome numerous host cell barriers to successfully traffic from the cell surface to the nucleus for genome delivery. Indeed, most of the incoming virions fail to follow this complex infectious route, accounting for the high particle-to-infectious ratio typically observed for PtPV infections. Therefore, productive and non-productive pathways are intermingled and hard to distinguish, which complicates the identification of the critical mechanisms leading to a productive infection. Nevertheless, the major steps of the entry process can be drawn faithfully. PtPVs enter the cells through receptor-mediated endocytosis using a wide variety of receptors, which explains their differences in tropism and pathogenesis. Subsequent to virus internalization, PtPVs enter the endocytic route. However, the timing of virus trafficking through the endosomal pathway, the cellular elements involved and the site of endosomal escape into the cytosol, vary considerably among virus species and host cells. Following endosomal escape a small subset of virions is transported to the nuclear vicinity, where delivery of the genome into the host nucleus takes place by as yet poorly characterized mechanisms.

This review is focused on the virus–host cell interactions involved during PtPV entry. The reader is referred to previous reviews on this issue [21,22,23,24]. Here, we have updated and summarized our current knowledge, and further discussed new aspects on entry mechanisms not previously covered. Starting from the initial interaction with host cell receptors, we guide through the sequential steps leading to delivery of the PtPV genome into the nucleus. The review is intended to provide a comprehensive view of the diversity of processes implicated in PtPV entry, including virus-cell interactions, sequential exposure of structural protein domains and capsid rearrangements. A detailed understanding of the molecular mechanisms underlying these processes will be pivotal to the rational design of PtPV vectors with enhanced cell entry properties, to establish efficient therapies against pathogenic PtPV for their control in nature, and to develop innovative PtPV-based anticancer therapies with improved efficiency and safety.

## 2. Functional Receptors for *Protoparvovirus* Entry

The initial interaction of parvoviruses with the host cell occurs upon binding to a specific receptor exposed on the host cell membrane. Recognition of cell surface molecules enables the first step of infection, and hence, represents a key parameter of tropism and pathogenesis. To date, different receptor biomolecules, such as proteins and carbohydrates with specific virus-binding properties, have been identified as attachment factors and, in some cases, as functional receptors for members of the PtPV genus. One of the best studied example is represented by the interaction of the transferrin receptor (TfR) with CPV and FPV. Both viruses use TfR to bind and infect cells, as the susceptibility to these viruses requires the constitutive or transient expression of the TfR [25]. Binding to TfR correlates with host specificity of these viruses, as FPV binds the feline TfR only, while CPV binds both the canine and feline TfRs. CPV variants accumulated changes on the top and side of the three-fold spike of the capsid surface, which confer more efficient use of the canine TfR receptor and reduce binding to the feline TfR [26,27].

**Figure 1:**
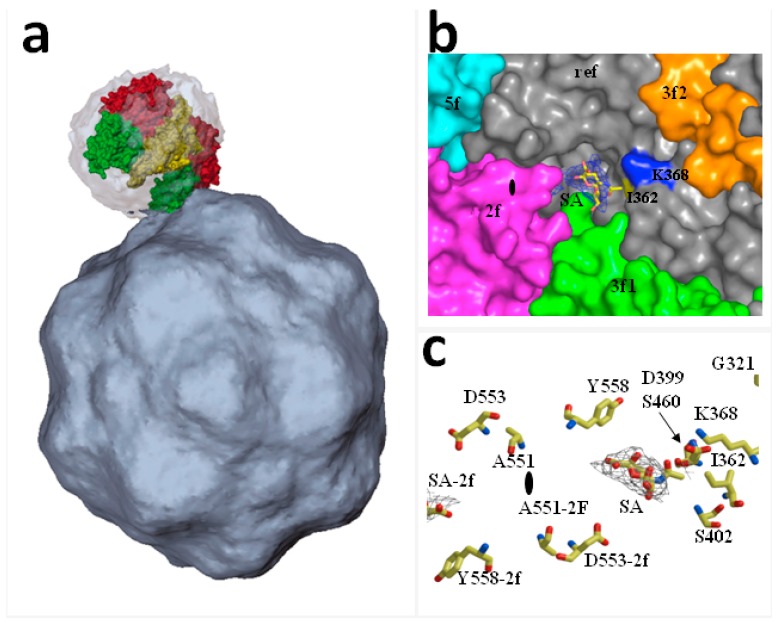
Structures of receptor-Protoparvovirus capsid complexes: (**a**) Asymmetric binding of transferrin receptor (TfR) to the CPV capsid. The image illustrates a TfR dimer manually fitted into the cryoEM density of CPV capsid. The interaction is best resolved between one of the two apical domains of the TfR dimer (green) and the shoulder of a spike near a three-fold icosahedral axis of the CPV capsid. (Reproduced with permission from Hafenstein, S. et al. [32]); (**b**,**c**) X-ray crystal structure identifying an infectious receptor attachment site on a PtPV capsid at high resolution: MVMp capsids soaked with sialic acid showing the electron density of the carbon backbone allocated in the twofold pocket adjacent to the I362 and K368 residues determining in vivo pathogenicity in severely combined immunodeficient (scid) mice. (Reproduced with ASM permission from Lopez-Bueno et al. [33]).

The receptor residues involved in CPV-specific binding localize to the TfR apical domain, and substitution of single amino acids may shift TfR binding ability to CPV or FPV [28,29]. The TfR molecules asymmetrically associated in vitro with a few of the sixty icosahedral equivalent binding sites on the CPV capsid near the three-fold axis of symmetry, Figure 1a. Importantly, the pattern of TfR-CPV capsid interaction was compromised by alteration of key residues of both the receptor and the virus capsid binding domain [30] such as mutations at residues 206–380 of the TfR apical domain which reduce binding to CPV capsids. In particular, the N-linked glycosylation site at position 384 in the canine TfR provided resistance to the carnivore parvoviruses circulating prior to about 1975, when a variant virus emerged that overcame this block [31]. The use of TfR receptors by CPV and FPV variants illustrates the capacity of PtPV to adapt to receptors determining host range.

Sialic acid (SA; *N*-acetylneuraminic acid) glycan isanother well-characterized PtPV receptor, which is probably the most commonly used in nature. Glycans represent major cell surface carbohydrate components, thereby providing a vast collection of cellular attachment factors for many viruses [34]. Biochemical studies have shown SA to serve as a common primary attachment factor for several PtPV. However, it is important to distinguish between unspecific attachment of parvoviral capsids to SA on multiple cell surfaces, and the functional interactions where SA acts as a receptor leading to the infection of permissive cells. For example, in vitro hemagglutination assays revealed that CPV and FPV bind to erythrocytes through SA, however the biological significance in the natural host infection, which is mediated by the TfRs (see above), remains unclear [35]. Similarly, the biological role(s) in the natural infection of the observed bovine parvovirus (BPV) binding to sialylated glycoproteins on erythrocyte and nucleated cell membranes [36,37,38] require further research. SA was first suggested to play a role in MVM binding to cell surfaces [3]. The virus binding sites per cell were in the range of 4–8 × 10^5^ SA molecules in established cell lines [39,40] and primary host cells [41]. Subsequently, it was demonstrated in cell culture experiments that indeed SA acts as functional receptor for the infection of several PtPV species, including MVM [33], PPV [42] and H1-PV [43].

PtPV/SA recognition plays a major role in the emergence of virulent viruses in vivo, as it was exemplified in adult severely combined immunodeficient (scid) mice infected by different MVM strains. The virulent immunosuppressive strain (MVMi) caused a severe leukopenia with deregulated erythropoiesis [44], whereas infections with the prototype MVM strain (MVMp) remained asymptomatic over several weeks [33]. However, upon eight weeks post-infection, lethal MVMp genetic variants emerged which carried only one of the three single amino acid changes (V325M, I362S, K368R) in the common sequence of their structural proteins. These mutations conferred lower capsid binding affinity to SA components of the natural receptor [33,41]. The substituted amino acid residues localize within a dimple depression at the icosahedral two-fold axis of symmetry in the MVM capsid, Figure 1b, and establish direct contact with the SA molecule, highlighting their role as crucial determinants of MVM in vivo pathogenicity and adaptation to the hosts.

Binding to an array of glycans enabled identification of SA types recognized by the two natural MVM strains, as well as by the emerged virulent capsid variants [45], and supported the evidences that lower affinity to SA glycans, rather than a different SA receptor specificity, determines MVM virulence. However, it remains unclear how capsid binding to SA-glycans may determine MVM tropism in vitro and in mice. Tropism determinants of MVMp and MVMi capsids in vitro mapped to a few residues which also localized to the two-fold dimple [46,47,48,49]. However, the *N*- and *O*-glycans and polar glycolipids composition of the cell lines used in those studies failed to explain MVM tropism solely based on the SA cell surface composition [50], suggesting the requirement of other cellular factors. MVMp undergoes a remarkable switch of tropism from asymptomatic fibrotropic to pathogenic hematotropic behavior upon weeks post- infection in scid mice [51]. This dramatic change in pathogenicity involved the emergence of a heterogeneous viral population harboring multiple genetic changes, which were strictly confined to the surface of the dimple surrounding the SA-receptor binding pocket [51]. Therefore, the capsid residues mediating direct contact with SA are major determinants of MVM virulence and tropism along its evolution in mice. Further research will be required to elucidate whether these genetically heterogeneous variants co-operate to cause disease, use different SA molecules to infect distinct hematopoietic precursors, or arise in naturally infected host settings.

## 3. Uptake into Host Cells

PtPVs are internalized by host cell receptor-mediated endocytosis upon binding to the functional receptor [22,23,52]. The endocytic route offers numerous advantages for the karyophilic parvoviruses. Endosomes may not only delay the detection of the incoming viral ssDNA by cellular sensors of innate immunity, but also provide a rapid and efficient transport towards the nuclear periphery, circumventing the viscous cytoplasm, where molecular crowding and the mesh-like structure of the cytoskeleton strongly hinder the mobility of macromolecules [53]. Exposure of incoming viruses to dynamic changes of the endocytic environment provides the triggers necessary to initiate the infection. The unique endolysosomal microenvironment differs considerably from the extracellular milieu [54]. The increasing acidic environment, changes in redox conditions and acid proteases and phosphatases, enable the capsids to undergo structural conformational changes required for the subsequent step of the infection, the endosomal escape.

There are distinct pinocytic pathways by which physiological cargo, as well as viral particles, can be taken into host cells. Clathrin- and caveolae-mediated endocytosis are two dynamin-dependent pathways. Macropinocytosis, lipid-raft mediated and the caveolin/clathrin-independent endocytosis are dynamin-independent pathways [55,56]. Clathrin-mediated endocytosis (CME) is the most common mechanism for endocytosis of small viruses and represents the default internalization route employed by parvoviruses [42,52,57,58,59]. A clathrin endocytic structure initiates upon clathrin recruitment to the plasma membrane by AP2 adaptor complexes. Clathrin forms a triskelion by the assembly of three heavy and three light chains to finally form a three-dimensional polygonal lattice, leading to progressive invagination of the plasma membrane into a clathrin-coated pit. Dynamin self-assembles at the neck of the coated pit and forms a collar-like structure that pinches off the clathrin-coated vesicle from the plasma membrane. The newly formed vesicle becomes rapidly uncoated and delivers its viral cargo by fusing with an early endosomal vesicle at the periphery of the cell [60].

Recent research has demonstrated that, in addition to the classical CME, PtPV may use several alternative endocytic routes. For example, PPV utilizes macropinocytosis [42] as additional uptake mechanism. CPV and FPV use TfR for uptake, which is typically endocytosed by CME. However, altering or removing the internalization signal of TfR delays uptake, but does not prevent infection [27], suggesting the existence of alternative uptake mechanisms. Most recently, it has been shown that MVMp enters its host cell by at least three potential endocytic routes [61]. Inhibition of various endocytic pathways with specific drugs in combination with electron microscopy (EM), immunofluorescence (IF) microscopy, and fluorescence-activated cell sorting (FACS), identified various endocytic pathways for MVM mediated by clathrin, caveolin, and clathrin-independent carriers (CLIC).However, the latter endocytic uptake mechanism was restricted to transformed cells only and did not occur in murine A9 fibroblasts. This observation was confirmed in further experiments which demonstrated that the dynamin inhibitor dynasore completely blocked MVM uptake in A9 mouse fibroblasts, whereas its inhibitory effect was incomplete in transformed cells [61]. These results indicate that both clathrin- and caveolin-mediated MVM endocytosis is dependent on dynamin in murine A9 fibroblasts, but transformed cells allow dynamin-independent CLIC-mediated uptake of MVMp. These observations imply that although internalization by clathrin might represent the main entry route of PtPV, other alternative routes of uptake might be involved in parallel or in certain cells.

Viruses have evolved different strategies to promote their internalization into host cells. Some viruses bind their cognate receptor(s) and remain immobile until they are endocytosed. Others, after binding their corresponding receptor will diffuse laterally at the cell surface seeking additional receptor molecules and inducing clustering of receptors prior to internalization. Following lateral diffusion along the plasma membrane, some particles are captured into preexisting or forming clathrin-coated areas, while others induce clathrin assembly at the site of binding [62,63]. Although the receptors utilized by some PtPVs have been defined in detail, the dynamics of virus-receptor(s) complex formation and the subsequent signaling cascade that triggers the uptake process remain poorly understood. The dynamics of the uptake process of PtPV have been best characterized for CPV. Upon binding to a limited number of TfRs, CPV capsids quickly diffuse laterally on the plasma membrane until they become captured by a forming clathrin coated pit [64]. The authors found a correlation between the number of receptors attached to a virus particle during lateral diffusion and the endocytic rate of these particles. Due to the low affinity of the virus capsids to TfR, the capsids can only diffuse during a short period of time on the cell surface. If not quickly captured by a pre-assembled clathrin coated pit, the capsids dissociate from its receptor. At present, it is not clear whether rodent PtPVs follow a similar uptake process involving lateral diffusion and capture by pre-existing clathrin structures. It is also unknown whether rodent PtPVs are readily internalized following the interaction with a single receptor, mainly SA, or require interactions with an additional receptor(s) to initiate the uptake process.

The oncotropism of rodent PtPV is influenced by multiple factors acting at different infection steps. It has recently been shown that one of those factors is an enhanced virus uptake in cancer cells. Cell migration, which is typically associated with some aggressive cancer cells, promotes MVMp uptake [65]. Another finding establishing a correlation between MVMp uptake and cancer cells is the requirement of MVMp for galectin-3, a multifunctional protein implicated in cancer metastasis. Galectin-3 may modulate clustering and signaling activity of the MVM receptor stimulating the uptake of the virus [66]. These results emphasize that, apart from factors occurring late during the infection, early events of the infection also contribute to the oncotropism of these viruses.

## 4. Endosomal Trafficking and Capsid Rearrangements

Endosomal trafficking of PtPV virions is thought to be a slow and rate-limiting process in viral infection [67]. The special endolysosomal microenvironment triggers crucial capsid structural rearrangements. For instance, MVM has been reported to traffic slowly through the endocytic pathway and only reaches the cell nucleus after 8 h post infection (hpi) when DNA replication was detected [68]. Several lines of evidence confirm that endosomal processing of incoming PtPV particles is essential. For CPV, capsids pre-treated at low pH were unable to accumulate in the nucleus following cytoplasmic injection [69]. For MVM, the structural rearrangements were equally impaired by lysosomotropic drugs, thereby preventing infection [67,68]. These drugs, such as bafilomycin A_1_ or the weak base chloroquine diphosphate, raise the endosomal pH by inhibiting the vacuolar-type H^+^ -adenosine triphosphatase (ATPase) [70,71], or by accumulating inside acidic compartments [72], respectively. Endosomal acidification has been demonstrated to be essential for the infection of CPV [57,69,73], MVM [67,68], as well as other parvoviruses.

The major rearrangements that PtPV capsids undertake along entry can be traced by the configuration and functions of the N-terminal sequences of the VP1 and VP2 subunits. The amino termini of VP1 (VP1-Nt, 1Nt or VP1uR) and VP2 (VP2-Nt or 2Nt) are flexible domains whose configuration cannot be resolved in the X-ray crystal structure of PtPV capsids [5,6,7], and are normally concealed in their interior. These sequences carry essential transport signals and are thought to translocate across the five-fold channel of the capsid. This led to the suggestion that VP N-termini act as drivers of the traffic of intact virions across the cellular compartments along the virus life cycle [12]. Disparate roles for these sequences along the entry of the virion inside the cell were first indirectly suggested as VP2 was found necessary for capsid assembly, but VP1 was essential for infectivity [74]. For VP1 this activity resided in functional domains of its unique terminal sequences (VP1uR), which included clusters of basic amino acids (BC1 to BC4) conserved in many parvoviruses [9], as well as other non-basic domains (e.g., PLA_2_, see below), which play specific roles during the entry process.

The externalization through the parvovirus fivefold capsid channel has been studied in detail for the VP2-Nt, as it can be fairly induced in vitro, although harsh conditions may also trigger VP1-Nt externalization in CPV [21,75], and MVM particles [76,77]. Assays conducted by multiple authors demonstrated that VP2-Nt externalization can be induced by heat in native capsids and virus like particles (VLPs), which for MVM share 3-D crystal structure [78]. Acidic treatment of CPV particles mimicking endosomal pH induced VP1-Nt exposure [79] but in MVM the cleavage of N-VP2 termini to VP3 was a prerequisite for VP1-Nt externalization [76,80]. VP2-Nt externalization was associated with a reproducible change in the emission fluorescence of tryptophan residues [81], which are located at distant positions to the channel [82], implying that heat provokes a wide conformational transition in the capsid. The dynamic exposure of Nt sequences across the channel is regulated by amino acid residues lying at the base of the pore [81], some of them configuring a hydrophobic gate tightly controlling the externalization of VP2-Nt and VP1 Nt sequences [80,83,84].

It is controversial to which extent these capsid structural rearrangements observed in vitro do occur in cells, and whether they play a role at early stages of the infection. Most evidence suggests that the in vitro treatments cannot recapitulate the complex physiological conditions in cells, implying that a combination of several factors, including receptor binding, low endosomal pH or interactions with unknown host proteins, are relevant for these structural transitions. However, in a few cases the in vivo observations support at least some of the in vitro structural changes. For example, as early as 30 min after endocytosis, the structural rearrangements of the MVM capsid allowed the cleavage of the exposed VP2-Nt, and the externalization of originally sequestered VP1-Nt, without the loss of capsid integrity [67].

**Figure 2:**
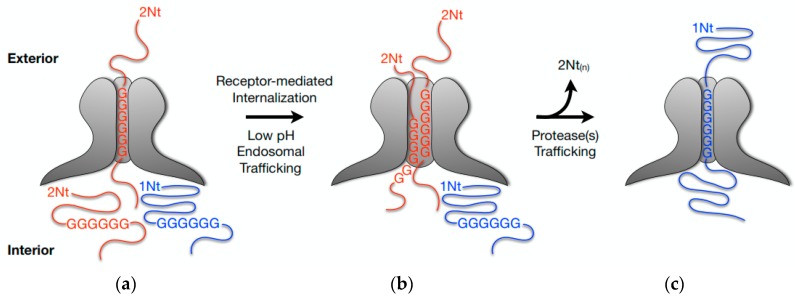
Model of the sequential externalization of VPs N-terminal sequences at the capsid pore during MVM entry: (**a**) In the extracellular virion the N-terminal sequence of VP2 subunits (VP2-Nt or 2Nt) are externalized through the capsid channel at the five-fold axis without impairing infectivity. One subunit anchored by the poly-Gly track is illustrated; (**b**) Upon receptor-mediated internalization and low pH exposure inside the endosome, internal VP2 N-termini access the base of the pore enlarging the functional diameter of the channel as they become extruded to the capsid exterior; (**c**) Most de novo exposed VP2 N-termini are cleaved-off from the capsid surface. The incoming virion traffics to downstream endosomal compartment(s), with presumed lower protease pools, where the VP1 N-terminus (or 1Nt) is entirely externalized becoming anchored by its poly-Gly track, as the channel returns to a narrow configuration. 1Nt (or VP1-Nt), the VP1-unique N-terminal sequence (143 amino acids); 2Nt (or VP2-Nt), the VP2 N-terminal sequence (27 amino acids); GGGG, poly-Glycine track. Based on references [82,91].

The complexity of the functions mediated by VP1-Nt and VP2-Nt during parvovirus entry is illustrated by the elusive role of the VP2-Nt in this process. The VP2-Nt domain may be cleaved in vitro to form the VP3 protein by a chymotrypsin-like protease [85,86,87,88]. In vivo, the VP2 to VP3 parvoviral cleavage is not a proteolytic maturation event as found in other non-enveloped viruses (e.g., Poliovirus [89]). It rather occurs in the pH-dependent entry pathway [24,42,68,75], soon after PtPV internalization [67]. Whether this cleavage plays any role in the infection was unclear since it could not be prevented by mutagenesis [86] or protease inhibitors [23]. Further, viral stocks exhibiting variable VP2/VP3 ratios [85,90] or even lacking VP3 subunits [12] were equally infectious. The genetic insertion of an exogenous peptide at VP2-Nt, however, inhibited VP2 cleavage and MVM infectivity, and it showed that neither VP3 nor VP1 subunits can accomplish the essential entry function(s) harbored by the VP2-Nt domain [91]. Although the exact role of the VP2-Nt in virus entry remains uncertain, an efficient exposure of the VP2 subunits through the channel, which is facilitated by the slender poly-Gly track [82], and their cleavage inside the cells, were necessary for the infection [91]. Therefore, we propose a role of the short VP2-Nt sequence on enlarging the functional diameter of the viral five-fold pore inside the endosome, determining the sequential exposure of VP2-Nt and VP1-Nt in the incoming metastable PtPV virion, Figure 2.

## 5. Endosomal Escape

Unlike enveloped viruses, which deliver their genomes into the host cell by fusion with the plasma or endosomal membranes [92], the non-enveloped viruses must employ alternative strategies to penetrate, disrupt or breach, the host cell’s delimiting membrane during entry. One of the most common mechanisms used for this purpose is the proteolytic cleavage of the viral particle [93]. In some cases an in vitro cleavage by a protease may activate the virus prior to cell inoculation [94], whereas in other virus systems a lytic peptide with endosomal membrane-interacting activity must be released from the virion inside the cell for full activity [95,96].

For PtPV the endosomal escape depends on the profound capsid structural rearrangements occurring inside the endosome (see above), which lead to the externalization of the disordered VP1-Nt. This VP1 sequence harbours a lipolytic activity to penetrate the endosomal membrane [8]. Evidences on the capacity of PtPV to permeabilize endosomal membranes came from the progressive release of labeled dextrans (MW 3 kDa) into the cytosol 8–20 h after co-endocytosis with CPV virions. Larger dextrans (MW 10 kDa) and α-sarcin were mainly retained in endosomes [57], suggesting incomplete disintegration of the endosomal membrane.

**Figure 3:**
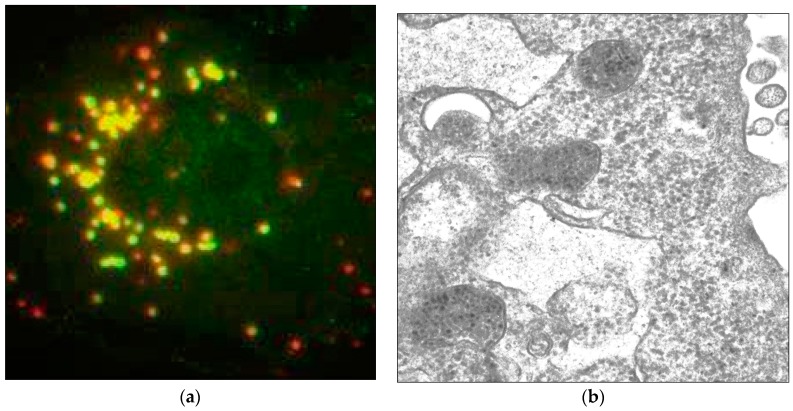
Accumulation and retention of incoming MVMp inside lysosomes: (**a**) Immunofluorescence picture taken 4 h post-infection from a single A9 cell infected with MVMp. Lysosomes are labeled in red (Lamp1) and viral capsids are labeled in green (B7 Mab) [97]; (**b**) Electron micrograph taken 18 h post-infection from an A9 cell infected with MVMp. A large amount of incoming viral particles is retained inside lysosomes.

However, the endosomal escape represents a major barrier for successful PtPV infection. Taking MVM as an example, a substantial proportion of the incoming MVM virions was demonstrated to follow a non-infectious pathway ending up in lysosomal compartments where they co-localized with lysosomal markers [67]. Hence, the endosomal escape represents the major barrier for the subsequent steps of MVM infection, Figure 3. However, the inability to escape from the endocytic route was not due to a failure in endosomal processing of MVM since all virions retained in lysosomal compartments underwent the required structural transitions. MVM VLPs or empty capsids (EC) that accumulated in lysosomes remained intact up to 50 hpi but the exposed viral DNA of full-virus capsids (FC) was degraded 21 hpi, most probably by the lysosomal endonuclease DNase II activity [67].

The mechanism of the PLA_2_ active domain which is harbored within VP1uR of the PtPV capsid and mediates endosomal escape has been extensively studied. The VP1-Nt becomes exposed in early endocytic vesicles [8,11,67]. Pre-incubation of VP1-Nt exposing CPV virions with PLA_2_ inhibitors, such as quinacrine and manoalide, significantly reduced or completely abolished infectivity, respectively [98]. For MVM complementation assays between wild-type and mutant particles have been used to demonstrate the role of PLA_2_ in mediating phospholipid bilayer penetration [11]. Accordingly, mutants with amino acid substitutions within their catalytic dyad severely impaired the enzymatic activity of the lipolytic PLA_2_, and viral infectivity was completely abrogated. Polyethyleneimine-induced endosomal rupture or co-infection with wild-type or mutant virions could partially rescue the mutant phenotype. Similarly, co-infection with endosomolytically active adenoviral variants resulted in a partial complementation of the mutant phenotype. Contrarily, endosomolytically inactive adenoviral variants, as well as wild-type EC carrying sequestered VP1uR sequences, were unable to restore infectivity of the PLA_2_-negative mutants. Thus, the capsid-tethered PLA_2_ motif seems to be either directly or indirectly required for successful penetration of the endosomal membranes.

The information regarding the precise site of PtPV endosomal escape is still incomplete. Previous in vitro experiments showed that the optimal pH for the parvoviral PLA_2_ enzymatic activity ranges between pH 6 to 7, but this activity drastically decreases at a pH below 5 [99]. Correspondingly, the acidic lysosomal environment would not provide optimal conditions for PLA_2_-mediated escape from the degradative pathway. Therefore, it is tempting to speculate that only a subset of incoming viruses manages to escape the endocytic route from a pre-lysosomal compartment in the absence of vesicle disintegration. This hypothesis is supported by the fact that MVM externalizes VP1-Nt already within the first minutes of infection, thus exposing the functional PLA_2_ enzymatic activity on its surface. Additionally, brefeldin A, an antibiotic blocking the transition between early and late endosomes has been demonstrated to abrogate MVM infection [68]. In summary, these results collectively suggest that a minority of virions that enter the cytosol escape from an intermediate pre-lysosomal vesicle, namely late endosomes [67].

Finally, it should be emphasized that understanding the infectious pathway of parvoviruses is complicated by the fact that the bulk of incoming particles are retained within lysosomal compartments following a degradative route. This phenomenon may largely account for the high particle-to-infection ratio found in most parvovirus–cell systems. Moreover, the lack of dynamic information in IF or EM experiments has complicated the study of virus trafficking through the highly dynamic and overlapping vesicular endocytic pathway. Emerging advances in time-lapse microscopy may render live-cell imaging an important complementary method to study the complex nature of PtPV endosomal trafficking.

## 6. Trafficking and Interactions in the Cytosol

Transport and maturation of incoming PtPV particles inside endosomes is not only required for endosomal escape, but also to confer transport and nuclear targeting capacity to the capsids in the cytoplasm. Capsids directly microinjected into the cytoplasm, bypassing endosomal maturation, remain immobile and fail to enter the nucleus [69,100]. However, capsid modifications continue also after endosomal escape. In general, it is difficult to examine PtPV capsids after endosomal escape, as most of the incoming capsids are retained in the endosomal pathway for many hours after uptake and do not actually contribute to the infection, Figure 3. The inefficient escape from endocytic vesicles represent a major complication to identify and characterize the particles that, following the infectious pathway, have reached the cytosol and target the nucleus for replication.

### 6.1. Interactions with the Cell Cytoskeleton and Motor Proteins

Following their release into the cytosol, the incoming viral particles are transported to the perinuclear area and enter the nucleus for replication. Cytoplasmic components and the intricate cytoskeleton network (microtubules (MTs), actin and intermediate filament) create a viscous cytosolic environment, with an average pore size of 20–40 nm [101]. These physical properties of the cytoplasm restrict the free diffusion of particles larger than 500 kDa or above 25 nm diameter [53,102,103,104,105]. The diameter of the parvovirus particle is close to the size limit that prohibits passive diffusion. Therefore, the efficient movement of parvovirus particles across the cytoplasm towards the nuclear vicinity is expected to mostly depend on microtubules and their associated motor proteins.

MTs are polarized structures with a plus end toward the cell periphery and a minus end toward the MT organizing center (MTOC) in the nuclear vicinity. Several studies have shown that PtPV infection requires intact MTs [42,68,69,79,106,107]. CPV has been demonstrated to depend on active, dynein-mediated retrograde transport along the MT. Nocodazole (ND), a highly specific drug promoting tubulin depolymerization in mammalian cells, prevented nuclear translocation of CPV. Similarly, an antibody against the intermediate chain of the motor protein dynein also reduced the nuclear accumulation of CPV capsids. EM based studies and co-immunoprecipitation (IP) of CPV with the intermediate chain of dynein supported a dynein-mediated transport of CPV along the microtubules toward the nucleus [79,106,107]. Similar observations were reported for MVM, where disruption of MT and actin filaments substantially reduced the infection [68].

Therefore, PtPV particles require the cytoskeleton and associated motor proteins to assist their transport to the nucleus. However, it is unclear whether the cytoskeleton is required to maintain the movement of the virus-containing endosomal vesicles or if a direct interaction of the incoming particles and cytoskeleton components exist in the cytosol. At least for CPV, it has been shown by IP and IF experiments, that capsids interact directly with dynein, the minus-end directed motor protein that transports cargo along MTs towards the MTOC near the nucleus [79]. The movement of cellular cargoes, as well as viruses, along MTs has been shown to be bi-directional and saltatory, involving cytoplasmic dynein (retrograde transport) and kinesin (anterograde transport) [108]. Accordingly, the association of incoming parvovirus particles with kinesin can be expected. Although not well understood, the net direction toward the nucleus is achieved during entry.

Apart from MTs and actin, the intermediate filament network (IFN) also plays an important role during viral infections [109]. The IFN protein vimentin is required by the incoming MVM particles during the endocytic trafficking and after endosomal escape in order to reach the perinuclear area. MVM infection induces the re-distribution of vimentin to perinuclear regions and MVM replication is reduced in cells without a functional vimentin IFN [110].

Collectively, these studies suggest that the cytoplasmic trafficking of PtPV capsids depends on the cell cytoskeleton, notably the MTs and associated motor proteins. However, the role of the cytoskeleton is not limited to the entry step but has been also implicated in late stages of the infection during virus egress. MVM transport from the nucleus to the cell periphery is associated with the degradation of actin fibers and the stabilization of microtubules [111]. Given the importance of MTs in many cellular processes and pathological disorders, including cancer, a thorough knowledge of the dynamic interactions of PtPV with the host cell transport machinery will be useful to better understand their infection mechanism and oncolytic properties.

### 6.2. Interactions with the Ubiquitin-Proteasome Machinery

The proteasome is a large multisubunit complex that selectively degrades ubiquitinated proteins. It is involved in a wide variety of proteolytically mediated intracellular processes, such as cell cycle progression, apoptosis, metabolic regulation and transcriptional control. The proteasome is also involved in the processing of antigens, generating peptides for the major histocompatibility complex pathway [112], and viruses from different families require proteasome activities in order to infect [113]. The ubiquitin-proteasome may also play important roles during PtPV entry, but current accumulated evidence is still mostly indirect. For example, although MVM and CPV capsids are not ubiquitinated during entry, these viruses, as well as PPV, require the activity of the proteasome for the infection [109]. In the presence of proteasome inhibitors, MVM capsids accumulate and persist in lysosomes and the infection is inhibited [68,114]. Since the proteasome control multiple biological processes, the identification of the specific step(s) of the PtPV infection where the proteasome activity is important, will be challenging.

### 6.3. Other Interactions in the Cytoplasm

Sumoylation is a post-translational modification analogous to ubiquitination, which involves addition of small ubiquitin-like modifiers (SUMOs). Sumoylation can affect protein stability, subcellular localization and protein–protein interactions. Putative roles of SUMO-PtPV interactions in entry are unknown, and therefore constitute promising issues for research.

Other cytoplasmic interactions, occurring during PtPV entry, may provoke cytotoxic effects associated with the successful infection. For example, the direct interaction of CPV with mitochondria during entry causes depolarization of the mitochondrial transmembrane potential and the induction of oxidative stress. These effects activate cell survival signaling through the ERK1/2 cascade and cell death [115].

## 7. Nuclear Entry and Uncoating

PtPV, as most DNA viruses, replicate within the cell nucleus making extensive use of the transcription and replication machineries of the host cells. PtPV structural proteins enter the nucleus at two stages of the PtPV life cycle; early in the infection, when the incoming virion delivers its genome for replication, and later when the newly expressed viral structural subunits are imported into the nucleus as assembly intermediates [16,116]. Macromolecules can translocate bi-directionally through the nuclear pore complex (NPC) via either passive or facilitated processes. While passive diffusion is related to ions and small to middle-size molecules, facilitated diffusion is a highly selective process mainly mediated by nuclear localization signals (NLS) exposed on the cargo molecules and recognized by specialized transport proteins [117,118]. The NPC has a functional diameter up to a maximum of 39 nm [119]. Larger macromolecules may still be translocated across the NPC, but must undergo rearrangements to reduce their diameter [118]. The functional diameter of the NPC central channel is in the range of the diameter of the parvovirus capsids. Therefore, unlike larger viruses, which partially or completely uncoat prior translocation through the NPC [120], the incoming parvovirus particle could pass through without disassembly.

Two distinct mechanisms have been proposed for the nuclear entry of incoming PtPV. The first body of data suggests that PtPV particles translocate across the NPC mediated by exposed NLS in the VP1 unique region (VP1uR). As mentioned above, PtPV undergo structural rearrangements triggered by the acidic conditions inside endosomes, resulting in the exposure of VP1uR. Cytoplasmic microinjection of VP1uR-specific antibodies was able to neutralize CPV infection [107]. Besides the PLA_2_ motif, which is required for endosomal escape (see above), VP1uR contains clusters of basic amino acids (BC) resembling classical NLS [9,121,122]. The BC1 sequence corresponds to a classical NLS, which commonly bind α and/or β importin receptors to translocate cargo across the NPC [123], and it was required for the nuclear translocation of newly synthesized VP1 subunits and VP1/VP2 complexes of MVM devoid of the VP2 nuclear transport motif [124]. Mutations in other BC sequences of VP1uR affected nuclear import of viral proteins and the infection, suggesting that they function in concert to facilitate the nuclear targeting of PtPV [9,122]. Accordingly, these data strongly suggest that the incoming PtPVs recruit the cellular import machinery to translocate across the NPC.

Other studies suggest a disparate NPC-independent nuclear entry following local disintegration of the nuclear membrane [125]. When microinjected into the cytoplasm of *Xenopus oocytes*, MVM was able to cause local nuclear envelope breakdown (NEB) in a time- and concentration-dependent manner [126]. It has been proposed that MVM hijacks a cellular pathway to disrupt the nuclear envelope of the host cell. The exact mechanism is not well understood, but appears to involve the re-localization of caspase 3 from the cytoplasm to the nucleus without its activation above basal levels in MVM infected cells. In the nucleus, caspase 3 cleaves lamin B2, resulting in a sustained disruption of the nuclear lamina structure and progression of nuclear envelope rupture. MVM-mediated, non-apoptotic caspase 3 activity induces nuclear entry of MVM capsids and possibly the nuclear targeting of further accessory proteins required for replication. Inhibition of caspase 3 during MVM infection resulted in a significant reduction of nuclear entry of MVM capsids and delayed expression of early viral gene products [127], hence the sequential molecular events leading to NEB were elucidated. The initial interaction of incoming capsids with NPC proteins would cause capsid structural rearrangements, which initiate a signal cascade resulting in local nuclear envelope disintegration. It was shown that calcium-mediated activation of Protein Kinase C (PKC) is essential for NEB, which together with caspase 3, triggers activation of the kinase cdk-2, a critical element governing the structure of the nuclear envelope [128]. These apparently contradictory observations may function in concert to promote PtPV nuclear entry. An initial docking of the PtPV capsids to the NPC could trigger a local disintegration of the nuclear envelope, which would facilitate the transport of the capsids across the NPC.

Finally, the mechanism and intracellular site of PtPV uncoating is not well understood. There are indications that PtPV capsids remain assembled in the cytosol and enter the nucleus intact. For example, CPV infectivity can be blocked by injecting neutralizing antibodies against intact capsids in the cytosol and nucleus, respectively [106]. The requirement of a complete disassembly to release PtPV DNA seems unlikely, and the viral DNA is more likely extruded from intact capsids. In vitro, the viral ssDNA can be externalized by a variety of treatments, which do not cause capsid disintegration, and the 3′ terminal hairpin can act as template for the DNA polymerase [76,77,114]. During the uncoating process, the viral DNA is thought to pass through the five-fold axis pore of the capsid [129]. These findings suggest that the partially exposed viral DNA within the nucleus can be used as template for initiating DNA replication by the host cell DNA polymerase, whereby the remaining internal DNA can be completely pulled out without the need to disassemble the stable PtPV capsid. However, the exact time of uncoating (prior, during or after entry into the nucleus) remains a matter of debate. IF experiments, either with fluorescently labeled virus or with conformational antibodies against assembled particles, have shown that the capsid enters the nucleus intact, although other authors have shown that viruses enter the nucleus after partial or total disassembly in the cytosol or NPC. The difficulties in discriminating between the infectious and the non-infectious routes could explain these contradictory observations.

## 8. Conclusions and Outlook

**Figure 4:**
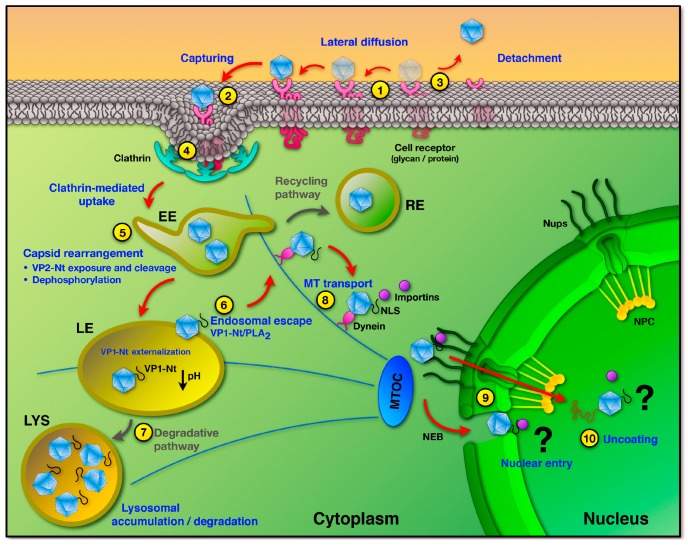
Model of PtPV cell entry and nuclear import. In the productive infection, PtPV capsids bind to one or more cell receptors (1). Following lateral diffusion, capsids are captured by pre-formed or forming clathrin pits (2). Or otherwise become detached from their receptors (3). Capsids are internalized via clathrin-mediated endocytosis (4) and enter the endocytic pathway where the low pH environment and local enzymes trigger structural rearrangements (5). Major structural changes are de novo VP2-Nt exposure and cleavage and particle dephosphorylation, leading to VP1-Nt (or VP1uR) exposure through the channel. The endosomal trafficking may vary in complexity and time, but commonly only a small proportion of particles escape after altering the endosomal membrane through the activity of the VP1-encoded phospholipase A2 (6). Many incoming infectious particles fail to escape from endosomes and are routed to and accumulated in the degradative lysosomes (7). In the cytosol, PtPV exploit the cytoskeleton and motor proteins to move to the nuclear vicinity (8). It is not yet clear how the viruses and/or their genomes enter the nucleus, as nuclear pore-dependent entry and permeabilization of the nuclear envelope have been proposed (9). The site and timing of capsid uncoating are also uncertain processes. Growing evidences suggest that capsids remain assembled in the cytosol and enter the nucleus intact, and the uncoating would take place during the interaction with NPC proteins and/or following nuclear entry (10).

This review was intended to update and summarize our current knowledge on the traffic of the *Protoparvovirus* infectious particle from the host cell plasma membrane to the nucleus. The study of this process is complex, because PtPV can use various pathways when infecting a cell, each one consisting of a number of steps, and each step may involve a series of specific and sequential interactions. Figure 4 illustrates the best experimentally supported pathways and their major regulatory mechanisms.

Increasing the level of complexity is the fact that PtPV–host interactions are dynamic, and moreover, many of those interactions are actually irrelevant for productive infection. Therefore, many unresolved questions will need to be addressed in the future in order to achieve a comprehensive understanding of PtPV entry. Major steps of the PtPV entry requiring detailed study should include the role of co-receptors, the precise mechanism underlying endosomal escape, the structures and paths involved in nuclear entry, specific triggers and the site where the PtPV genome becomes accessible for replication. The complexity of these tasks demands the improvement of the current technology and the implementation of novel methods, such as the combination of single-virus tracking in living cells with high-resolution imaging technologies or large-scale RNAi screening. A better knowledge of the intricate process of PtPV entry will be fundamental, not only to better understand the tropism, host range and pathogenesis of the infections cause by pathogenic PtPVs, but also to improve and expand the application of non-pathogenic rodent parvoviruses in cancer therapy.

## References

[1] Cotmore S.F., Agbandje-McKenna M., Chiorini J.A., Mukha D.V., Pintel D.J., Qiu J., Soderlund-Venermo M., Tattersall P., Tijssen P., Gatherer D. (2014). The family parvoviridae. Arch. Virol..

[2] Kailasan S., Agbandje-McKenna M., Parrish C.R. (2015). Parvovirus family conundrum: What makes a killer?. Annu. Rev. Virol..

[3] Cotmore S.F., Tattersall P. (1987). The autonomously replicating parvoviruses of vertebrates. Adv. Virus Res..

[4] Tijssen P., Agbandje-McKenna M., Almendral J.M., Bergoin M., Flegel T.W., Hedman K., Kleinschmidt J., Li Y., Pintel D.J., Tattersall P., King A., Lefkowitz E., Adams M.J., Carstens E.B. (2012). Parvoviridae. Virus Taxonomy.

[5] Tsao J., Chapman M.S., Agbandje M., Keller W., Smith K., Wu H., Luo M., Smith T.J., Rossmann M.G., Compans R.W. (1991). The three-dimensional structure of canine parvovirus and its functional implications. Science.

[6] Agbandje-McKenna M., Llamas-Saiz A.L., Wang F., Tattersall P., Rossmann M.G. (1998). Functional implications of the structure of the murine parvovirus, minute virus of mice. Structure.

[7] Kontou M., Govindasamy L., Nam H.-J., Bryant N., Llamas-Saiz A.L., Foces-Foces C., Hernando E., Rubio M.-P., McKenna R., Almendral J.M. (2005). Structural determinants of tissue tropism and in vivo pathogenicity for the parvovirus minute virus of mice. J. Virol..

[8] Zádori Z., Szelei J., Lacoste M.C., Li Y., Gariépy S., Raymond P., Allaire M., Nabi I.R., Tijssen P. (2001). A viral phospholipase A2 is required for parvovirus infectivity. Dev. Cell.

[9] Lombardo E., Ramírez J.C., Garcia J., Almendral J.M. (2002). Complementary roles of multiple nuclear targeting signals in the capsid proteins of the parvovirus minute virus of mice during assembly and onset of infection. J. Virol..

[10] Vihinen-Ranta M., Wang D., Weichert W.S., Parrish C.R. (2002). The VP1 N-terminal sequence of canine parvovirus affects nuclear transport of capsids and efficient cell infection. J. Virol..

[11] Farr G.A., Zhang L.-G., Tattersall P. (2005). Parvoviral virions deploy a capsid-tethered lipolytic enzyme to breach the endosomal membrane during cell entry. Proc. Natl. Acad. Sci. USA.

[12] Maroto B., Valle N., Saffrich R., Almendral J.M. (2004). Nuclear export of the nonenveloped parvovirus virion is directed by an unordered protein signal exposed on the capsid surface. J. Virol..

[13] Weitzman M.D., Kerr J.R., Cotmore S.F., Bloom M.E., Linden R.M., Parrish C.R. (2006). The parvovirus life cycle: An introduction to molecular interactions important for infection. Parvoviruses.

[14] Bashir T., Horlein R., Rommelaere J., Willwand K. (2000). Cyclin A activates the DNA polymerase delta—Dependent elongation machinery in vitro: A parvovirus DNA replication model. Proc. Natl. Acad. Sci. USA.

[15] Gil-Ranedo J., Hernando E., Riolobos L., Domínguez C., Kann M., Almendral J.M. (2015). The mammalian cell cycle regulates parvovirus nuclear capsid assembly. PLoS Pathog..

[16] Riolobos L., Valle N., Hernando E., Maroto B., Kann M., Almendral J.M. (2010). Viral oncolysis that targets Raf-1 signaling control of nuclear transport. J. Virol..

[17] Nüesch J.P.F., Lacroix J., Marchini A., Rommelaere J. (2012). Molecular pathways: Rodent parvoviruses—Mechanisms of oncolysis and prospects for clinical cancer treatment. Clin. Cancer Res..

[18] Ventoso I., Berlanga J.J., Almendral J.M. (2010). Translation control by protein kinase R restricts minute virus of mice infection: Role in parvovirus oncolysis. J. Virol..

[19] Angelova A.L., Geletneky K., Nüesch J.P.F., Rommelaere J. (2015). Tumor selectivity of oncolytic parvoviruses: From in vitro and animal models to cancer patients. Front. Bioeng. Biotechnol..

[20] Marchini A., Bonifati S., Scott E.M., Angelova A.L., Rommelaere J. (2015). Oncolytic parvoviruses: From basic virology to clinical applications. Virol. J..

[21] Vihinen-Ranta M., Suikkanen S., Parrish C.R. (2004). Pathways of cell infection by parvoviruses and adeno-associated viruses. J. Virol..

[22] Harbison C.E., Chiorini J.A., Parrish C.R. (2008). The parvovirus capsid odyssey: From the cell surface to the nucleus. Trends Microbiol..

[23] Cotmore S.F., Tattersall P. (2007). Parvoviral host range and cell entry mechanisms. Adv. Virus Res..

[24] Parrish C.R. (2010). Structures and functions of parvovirus capsids and the process of cell infection. Curr. Top. Microbiol. Immunol..

[25] Parker J.S.L., Murphy W.J., Wang D., O’Brien S.J., Parrish C.R. (2001). Canine and feline parvoviruses can use human or feline transferrin receptors to bind, enter, and infect cells. J. Virol..

[26] Hueffer K., Parker J.S.L., Weichert W.S., Geisel R.E., Sgro J.-Y., Parrish C.R. (2003). The natural host range shift and subsequent evolution of canine parvovirus resulted from virus-specific binding to the canine transferrin receptor. J. Virol..

[27] Hueffer K., Palermo L.M., Parrish C.R. (2004). Parvovirus infection of cells by using variants of the feline transferrin receptor altering clathrin-mediated endocytosis, membrane domain localization, and capsid-binding domains. J. Virol..

[28] Palermo L.M., Hueffer K., Parrish C.R. (2003). Residues in the apical domain of the feline and canine transferrin receptors control host-specific binding and cell infection of canine and feline parvoviruses. J. Virol..

[29] Palermo L.M., Hafenstein S.L., Parrish C.R. (2006). Purified feline and canine transferrin receptors reveal complex interactions with the capsids of canine and feline parvoviruses that correspond to their host ranges. J. Virol..

[30] Kaelber J.T., Demogines A., Harbison C.E., Allison A.B., Goodman L.B., Ortega A.N., Sawyer S.L., Parrish C.R. (2012). Evolutionary reconstructions of the transferrin receptor of Caniforms supports canine parvovirus being a re-emerged and not a novel pathogen in dogs. PLoS Pathog..

[31] Allison A.B., Kohler D.J., Ortega A., Hoover E.A., Grove D.M., Holmes E.C., Parrish C.R. (2014). Host-specific parvovirus evolution in nature is recapitulated by in vitro adaptation to different carnivore species. PLoS Pathog..

[32] Hafenstein S., Palermo L.M., Kostyuchenko V.A., Xiao C., Morais M.C., Nelson C.D.S., Bowman V.D., Battisti A.J., Chipman P.R., Parrish C.R. (2007). Asymmetric binding of transferrin receptor to parvovirus capsids. Proc. Natl. Acad. Sci. USA.

[33] Lopez-Bueno A., Rubio M.-P., Bryant N., McKenna R., Agbandje-McKenna M., Almendral J.M. (2006). Host-selected amino acid changes at the sialic acid binding pocket of the parvovirus capsid modulate cell binding affinity and determine virulence. J. Virol..

[34] Olofsson S., Bergström T. (2005). Glycoconjugate glycans as viral receptors. Ann. Med..

[35] Barbis D.P., Chang S.F., Parrish C.R. (1992). Mutations adjacent to the dimple of the canine parvovirus capsid structure affect sialic acid binding. Virology.

[36] Johnson F.B., Thacker T.C. (1998). Binding of bovine parvovirus to erythrocyte membrane sialylglycoproteins. J. Gen. Virol..

[37] Johnson F.B., Fenn L.B., Owens T.J., Faucheux L.J., Blackburn S.D. (2004). Attachment of bovine parvovirus to sialic acids on bovine cell membranes. J. Gen. Virol..

[38] Blackburn S.D., Cline S.E., Hemming J.P., Johnson F.B. (2005). Attachment of bovine parvovirus to O-linked α 2,3 neuraminic acid on glycophorin A. Arch. Virol..

[39] Linser P., Bruning H., Armentrout R.W. (1977). Specific binding sites for a parvovirus, minute virus of mice, on cultured mouse cells. J. Virol..

[40] Spalholz B.A., Tattersall P. (1983). Interaction of minute virus of mice with differentiated cells: strain-dependent target cell specificity is mediated by intracellular factors. J. Virol..

[41] Rubio M.-P., López-Bueno A., Almendral J.M. (2005). Virulent variants emerging in mice infected with the apathogenic prototype strain of the parvovirus minute virus of mice exhibit a capsid with low avidity for a primary receptor. J. Virol..

[42] Boisvert M., Fernandes S., Tijssen P. (2010). Multiple pathways involved in porcine parvovirus cellular entry and trafficking toward the nucleus. J. Virol..

[43] Allaume X., El-Andaloussi N., Leuchs B., Bonifati S., Kulkarni A., Marttila T., Kaufmann J.K., Nettelbeck D.M., Kleinschmidt J., Rommelaere J. (2012). Retargeting of rat parvovirus H-1PV to cancer cells through genetic engineering of the viral capsid. J. Virol..

[44] Segovia J.C., Gallego J.M., Bueren J.A., Almendral J.M. (1999). Severe leukopenia and dysregulated erythropoiesis in SCID mice persistently infected with the parvovirus minute virus of mice. J. Virol..

[45] Nam H.-J., Gurda-Whitaker B., Gan W.Y., Ilaria S., McKenna R., Mehta P., Alvarez R.A., Agbandje-McKenna M. (2006). Identification of the sialic acid structures recognized by minute virus of mice and the role of binding affinity in virulence adaptation. J. Biol. Chem..

[46] Antonietti J.P., Sahli R., Beard P., Hirt B. (1988). Characterization of the cell type-specific determinant in the genome of minute virus of mice. J. Virol..

[47] Ball-Goodrich L.J., Tattersall P. (1992). Two amino acid substitutions within the capsid are coordinately required for acquisition of fibrotropism by the lymphotropic strain of minute virus of mice. J. Virol..

[48] Gardiner E.M., Tattersall P. (1988). Mapping of the fibrotropic and lymphotropic host range determinants of the parvovirus minute virus of mice. J. Virol..

[49] Tattersall P., Bratton J. (1983). Reciprocal productive and restrictive virus-cell interactions of immunosuppressive and prototype strains of minute virus of mice. J. Virol..

[50] Halder S., Cotmore S., Heimburg-Molinaro J., Smith D.F., Cummings R.D., Chen X., Trollope A.J., North S.J., Haslam S.M., Dell A. (2014). Profiling of glycan receptors for minute virus of mice in permissive cell lines towards understanding the mechanism of cell recognition. PLoS ONE.

[51] Lopez-Bueno A., Segovia J.C., Bueren J.A., O’Sullivan M.G., Wang F., Tattersall P., Almendral J.M. (2008). Evolution to pathogenicity of the parvovirus minute virus of mice in immunodeficient mice involves genetic heterogeneity at the capsid domain that determines tropism. J. Virol..

[52] Dudleenamjil E., Lin C.-Y., Dredge D., Murray B.K., Robison R.A., Johnson F.B. (2010). Bovine parvovirus uses clathrin-mediated endocytosis for cell entry. J. Gen. Virol..

[53] Seksek O., Biwersi J., Verkman A.S. (1997). Translational diffusion of macromolecule-sized solutes in cytoplasm and nucleus. J. Cell Biol..

[54] Pillay C.S., Elliott E., Dennison C. (2002). Endolysosomal proteolysis and its regulation. Biochem. J..

[55] Doherty G.J., McMahon H.T. (2009). Mechanisms of endocytosis. Annu. Rev. Biochem..

[56] Mercer J., Schelhaas M., Helenius A. (2010). Virus entry by endocytosis. Annu. Rev. Biochem..

[57] Parker J.S., Parrish C.R. (2000). Cellular uptake and infection by canine parvovirus involves rapid dynamin-regulated clathrin-mediated endocytosis, followed by slower intracellular trafficking. J. Virol..

[58] Vendeville A., Ravallec M., Jousset F.-X., Devise M., Mutuel D., López-Ferber M., Fournier P., Dupressoir T., Ogliastro M. (2009). Densovirus infectious pathway requires clathrin-mediated endocytosis followed by trafficking to the nucleus. J. Virol..

[59] Quattrocchi S., Ruprecht N., Bönsch C., Bieli S., Zürcher C., Boller K., Kempf C., Ros C. (2012). Characterization of the early steps of human parvovirus B19 infection. J. Virol..

[60] McMahon H.T., Boucrot E. (2011). Molecular mechanism and physiological functions of clathrin-mediated endocytosis. Nat. Rev. Mol. Cell Biol..

[61] Garcin P.O., Panté N. (2015). The minute virus of mice exploits different endocytic pathways for cellular uptake. Virology.

[62] Alexander S., Puthenveedu M.A., Yarden Y., Tarcic G. (2013). Clathrin-mediated endocytosis. Vesicle Trafficking in Cancer.

[63] Boulant S., Stanifer M., Lozach P.-Y. (2015). Dynamics of virus-receptor interactions in virus binding, signaling, and endocytosis. Viruses.

[64] Cureton D.K., Harbison C.E., Cocucci E., Parrish C.R., Kirchhausen T. (2012). Limited transferrin receptor clustering allows rapid diffusion of canine parvovirus into clathrin endocytic structures. J. Virol..

[65] Garcin P.O., Panté N. (2014). Cell migration is another player of the minute virus of mice infection. Virology.

[66] Garcin P.O., Nabi I.R., Panté N. (2015). Galectin-3 plays a role in minute virus of mice infection. Virology.

[67] Mani B., Baltzer C., Valle N., Almendral J.M., Kempf C., Ros C. (2006). Low pH-dependent endosomal processing of the incoming parvovirus minute virus of mice virion leads to externalization of the VP1 N-terminal sequence (N-VP1), N-VP2 cleavage, and uncoating of the full-length genome. J. Virol..

[68] Ros C., Burckhardt C.J., Kempf C. (2002). Cytoplasmic trafficking of minute virus of mice: low-pH requirement, routing to late endosomes, and proteasome interaction. J. Virol..

[69] Vihinen-Ranta M., Kalela A., Mäkinen P., Kakkola L., Marjomäki V., Vuento M. (1998). Intracellular route of canine parvovirus entry. J. Virol..

[70] Bowman E.J., Siebers A., Altendorf K. (1988). Bafilomycins: a class of inhibitors of membrane ATPases from microorganisms, animal cells, and plant cells. Proc. Natl. Acad. Sci. USA.

[71] Hensens O.D., Monaghan R.L., Huang L., Albers-Schonberg G. (1983). Structure of the sodium and potassium ion activated adenosine triphosphatase inhibitor L-681,110. J. Am. Chem. Soc..

[72] Ohkuma S., Poole B. (1978). Fluorescence probe measurement of the intralysosomal pH in living cells and the perturbation of pH by various agents. Proc. Natl. Acad. Sci. USA.

[73] Basak S., Turner H. (1992). Infectious entry pathway for canine parvovirus. Virology.

[74] Tullis G.E., Burger L.R., Pintel D.J. (1993). The minor capsid protein VP1 of the autonomous parvovirus minute virus of mice is dispensable for encapsidation of progeny single-stranded DNA but is required for infectivity. J. Virol..

[75] Weichert W.S., Parker J.S.L., Wahid A.T.M., Chang S.-F., Meier E., Parrish C.R. (1998). Assaying for structural variation in the parvovirus capsid and its role in infection. Virology.

[76] Cotmore S.F., D’Abramo A.M., Ticknor C.M., Tattersall P. (1999). Controlled conformational transitions in the MVM virion expose the VP1 N-Terminus and viral genome without particle disassembly. Virology.

[77] Cotmore S.F., Hafenstein S., Tattersall P. (2010). Depletion of virion-associated divalent cations induces parvovirus minute virus of mice to eject its genome in a 3′-to-5′ direction from an otherwise intact viral particle. J. Virol..

[78] Hernando E., Llamas-Saiz A.L., Foces-Foces C., McKenna R., Portman I., Agbandje-McKenna M., Almendral J.M. (2000). Biochemical and physical characterization of parvovirus minute virus of mice virus-like particles. Virology.

[79] Suikkanen S., Aaltonen T., Nevalainen M., Välilehto O., Lindholm L., Vuento M., Vihinen-Ranta M. (2003). Exploitation of microtubule cytoskeleton and dynein during parvoviral traffic toward the nucleus. J. Virol..

[80] Farr G.A., Cotmore S.F., Tattersall P. (2006). VP2 cleavage and the leucine ring at the base of the fivefold cylinder control pH-dependent externalization of both the VP1 N terminus and the genome of minute virus of mice. J. Virol..

[81] Reguera J., Carreira A., Riolobos L., Almendral J.M., Mateu M.G. (2004). Role of interfacial amino acid residues in assembly, stability, and conformation of a spherical virus capsid. Proc. Natl. Acad. Sci. USA.

[82] Castellanos M., Pérez R., Rodríguez-Huete A., Grueso E., Almendral J.M.M., Mateu M.G. (2013). A slender tract of glycine residues is required for translocation of the VP2 protein N-terminal domain through the parvovirus MVM capsid channel to initiate infection. Biochem. J..

[83] Farr G.A., Tattersall P. (2004). A conserved leucine that constricts the pore through the capsid fivefold cylinder plays a central role in parvoviral infection. Virology.

[84] Subramanian S., Organtini L.J., Grossman A., Domeier P.P., Cifuente J.O., Makhov A.M., Conway J.F., D’Abramo A., Cotmore S.F., Tattersall P. (2017). Cryo-EM maps reveal five-fold channel structures and their modification by gatekeeper mutations in the parvovirus minute virus of mice (MVM) capsid. Virology.

[85] Tattersall P., Shatkin A.J., Ward D.C. (1977). Sequence homology between the structural polypeptides of minute virus of mice. J. Mol. Biol..

[86] Tullis G.E., Burger L.R., Pintel D.J. (1992). The trypsin-sensitive RVER domain in the capsid proteins of minute virus of mice is required for efficient cell binding and viral infection but not for proteolytic processing in vivo. Virology.

[87] Paradiso P.R., Williams K.R., Costantino R.L. (1984). Mapping of the amino terminus of the H-1 parvovirus major capsid protein. J. Virol..

[88] Paradiso P.R. (1981). Infectious process of the parvovirus H-1: Correlation of protein content, particle density, and viral infectivity. J. Virol..

[89] Vincent R., Knipe D., Howley P. (2001). Racaniello Fields Virology. Picornaviridae: The Viruses and Their Replication.

[90] Tattersall P., Cawte P.J., Shatkin A.J., Ward D.C. (1976). Three structural polypeptides coded for by minite virus of mice, a parvovirus. J. Virol..

[91] Sánchez-Martínez C., Grueso E., Carroll M., Rommelaere J., Almendral J.M. (2012). Essential role of the unordered VP2 n-terminal domain of the parvovirus MVM capsid in nuclear assembly and endosomal enlargement of the virion fivefold channel for cell entry. Virology.

[92] Harrison S.C. (2008). Viral membrane fusion. Nat. Struct. Mol. Biol..

[93] Tsai B. (2007). Penetration of nonenveloped viruses into the cytoplasm. Annu. Rev. Cell Dev. Biol..

[94] Richards R.M., Lowy D.R., Schiller J.T., Day P.M. (2006). Cleavage of the papillomavirus minor capsid protein, L2, at a furin consensus site is necessary for infection. Proc. Natl. Acad. Sci. USA.

[95] Odegard A.L., Kwan M.H., Walukiewicz H.E., Banerjee M., Schneemann A., Johnson J.E. (2009). Low endocytic pH and capsid protein autocleavage are critical components of Flock House virus cell entry. J. Virol..

[96] Chandran K., Farsetta D.L., Nibert M.L. (2002). Strategy for nonenveloped virus entry: A hydrophobic conformer of the reovirus membrane penetration protein micro 1 mediates membrane disruption. J. Virol..

[97] Kaufmann B., López-Bueno A., Mateu M.G., Chipman P.R., Nelson C.D.S., Parrish C.R., Almendral J.M., Rossmann M.G. (2007). Minute virus of mice, a parvovirus, in complex with the Fab fragment of a neutralizing monoclonal antibody. J. Virol..

[98] Suikkanen S., Antila M., Jaatinen A., Vihinen-Ranta M., Vuento M. (2003). Release of canine parvovirus from endocytic vesicles. Virology.

[99] Canaan S., Zádori Z., Ghomashchi F., Bollinger J., Sadilek M., Moreau M.E., Tijssen P., Gelb M.H. (2004). Interfacial enzymology of parvovirus phospholipases A2. J. Biol. Chem..

[100] Lyi S.M., Tan M.J.A., Parrish C.R. (2014). Parvovirus particles and movement in the cellular cytoplasm and effects of the cytoskeleton. Virology.

[101] Luby-Phelps K., Taylor D.L., Lanni F. (1986). Probing the structure of cytoplasm. J. Cell Biol..

[102] Luby-Phelps K. (1999). Cytoarchitecture and physical properties of cytoplasm: Volume, viscosity, diffusion, intracellular surface area. Int. Rev. Cytol..

[103] Lagache T., Dauty E., Holcman D. (2009). Physical principles and models describing intracellular virus particle dynamics. Curr. Opin. Microbiol..

[104] Novak I.L., Kraikivski P., Slepchenko B.M. (2009). Diffusion in cytoplasm: Effects of excluded volume due to internal membranes and cytoskeletal structures. Biophys. J..

[105] Wirtz D. (2009). Particle-tracking microrheology of living cells: Principles and applications. Annu. Rev. Biophys..

[106] Vihinen-Ranta M., Yuan W., Parrish C.R. (2000). Cytoplasmic trafficking of the canine parvovirus capsid and its role in infection and nuclear transport. J. Virol..

[107] Suikkanen S., Sääjärvi K., Hirsimäki J., Välilehto O., Reunanen H., Vihinen-Ranta M., Vuento M. (2002). Role of recycling endosomes and lysosomes in dynein-dependent entry of canine parvovirus. J. Virol..

[108] Welte M.A. (2004). Bidirectional transport along microtubules. Curr. Biol..

[109] Sripada S., Dayaraj C. (2010). Viral interactions with intermediate filaments: Paths less explored. Cell Health Cytoskelet..

[110] Fay N., Panté N. (2013). The intermediate filament network protein, vimentin, is required for parvoviral infection. Virology.

[111] Nüesch J.P.F.F., Lachmann S., Rommelaere J. (2005). Selective alterations of the host cell architecture upon infection with parvovirus minute virus of mice. Virology.

[112] Bedford L., Paine S., Sheppard P.W., Mayer R.J., Roelofs J. (2010). Assembly, structure, and function of the 26S proteasome. Trends Cell Biol..

[113] Luo H. (2016). Interplay between the virus and the ubiquitin–proteasome system: Molecular mechanism of viral pathogenesis. Curr. Opin. Virol..

[114] Ros C., Kempf C. (2004). The ubiquitin-proteasome machinery is essential for nuclear translocation of incoming minute virus of mice. Virology.

[115] Nykky J., Vuento M., Gilbert L. (2014). Role of mitochondria in parvovirus pathology. PLoS ONE.

[116] Riolobos L., Reguera J., Mateu M.G., Almendral J.M. (2006). Nuclear transport of trimeric assembly intermediates exerts a morphogenetic control on the icosahedral parvovirus capsid. J. Mol. Biol..

[117] Timney B.L., Raveh B., Mironska R., Trivedi J.M., Kim S.J., Russel D., Wente S.R., Sali A., Rout M.P. (2016). Simple rules for passive diffusion through the nuclear pore complex. J. Cell Biol..

[118] Wente S.R., Rout M.P. (2010). The nuclear pore complex and nuclear transport. Cold Spring Harb. Perspect. Biol..

[119] Panté N., Kann M. (2002). Nuclear pore complex is able to transport macromolecules with diameters of about 39 nm. Mol. Biol. Cell.

[120] Fay N., Panté N. (2015). Nuclear entry of DNA viruses. Front. Microbiol..

[121] Vihinen-Ranta M., Kakkola L., Kalela A., Vilja P., Vuento M. (1997). Characterization of a nuclear localization signal of canine parvovirus capsid proteins. Eur. J. Biochem..

[122] Boisvert M., Bouchard-Lévesque V., Fernandes S., Tijssen P. (2014). Classic nuclear localization signals and a novel nuclear localization motif are required for nuclear transport of porcine parvovirus capsid proteins. J. Virol..

[123] Lange A., Mills R.E., Lange C.J., Stewart M., Devine S.E., Corbett A.H. (2007). Classical nuclear localization signals: Definition, function, and interaction with importin α. J. Biol. Chem..

[124] Lombardo E., Ramírez J.C., Agbandje-McKenna M., Almendral J.M. (2000). A β-stranded motif drives capsid protein oligomers of the parvovirus minute virus of mice into the nucleus for viral assembly. J. Virol..

[125] Cohen S., Behzad A.R., Carroll J.B., Panté N., Pante N. (2006). Parvoviral nuclear import: Bypassing the host nuclear-transport machinery. J. Gen. Virol..

[126] Cohen S., Panté N. (2005). Pushing the envelope: Microinjection of Minute virus of mice into Xenopus oocytes causes damage to the nuclear envelope. J. Gen. Virol..

[127] Cohen S., Marr A.K., Garcin P., Pante N., Panté N. (2011). Nuclear Envelope Disruption Involving Host Caspases Plays a Role in the Parvovirus Replication Cycle. J. Virol..

[128] Porwal M., Cohen S., Snoussi K., Popa-Wagner R., Anderson F., Dugot-Senant N., Wodrich H., Dinsart C., Kleinschmidt J.A., Panté N. (2013). Parvoviruses cause nuclear envelope breakdown by activating key enzymes of mitosis. PLoS Pathog..

[129] Cotmore S.F., Tattersall P. (2012). Mutations at the base of the icosahedral five-fold cylinders of minute virus of mice induce 3′-to-5′ genome uncoating and critically impair entry functions. J. Virol..

